# Linking co-expression modules with phenotypes

**DOI:** 10.6026/97320630018438

**Published:** 2022-04-30

**Authors:** Rakesh Kumar, Krishna Kumar Ojha, Harlokesh Narayan Yadav, Vijay Kumar Singh

**Affiliations:** 1 Department of Bioinformatics, Central University of South Bihar, Gaya, Bihar 824236, India; 2 Department of Pharmacology, All India Institute of Medical Sciences, Ansari Nagar, New Delhi - 110029, India

**Keywords:** Analysis of rank, co-expression, gene module, gene expression, network

## Abstract

The method for quantifying the association between co-expression module and clinical trait of interest requires application of dimensionality reduction to summaries modules as one dimensional (1D) vector. However, these methods are often linked with information loss. The amount of information lost depends upon the percentage of variance captured by the reduced 1D vector. Therefore, it is of interest to describe a method using analysis of rank (AOR) to assess the association between module and clinical trait of interest. This method works with clinical traits represented as binary class labels and can be adopted for clinical traits measured in continuous scale by dividing samples in two groups around median value. Application of the AOR method on test data for muscle gene expression profiles identifies modules significantly associated with diabetes status.

## Background:

In recent years the transcriptomic data analysis has witness a shift from gene level analysis to gene modules level analysis [1,2]. Module level analysis aims to identify set of co-expressed/co-regulated genes from transcriptomic data [3,4]. Further downstream analysis includes identification of intra-modular hubs, investigation of relationship between co-expression modules and the comparison of network topology of different networks [5, 6]. Generally module as whole is summarized with either meta-gene representing average expression of genes for each sample or with module eigen genes which can be considered as the best summary of standardized module expression data [7]. The module eigen gene of a given module is defined as the first principal component of the standardized expression profiles [8]. To find modules that relate to a clinical trait of interest, the module eigen genes are correlated with the clinical trait of interest [9,10]. Therefore, it is of interest to describe a method using analysis of rank (AOR) to assess the association between module and clinical trait of interest.

## Materials and methods:

### The AOR algorithm to assess module-trait association:

The method works such that where clinical traits are distinct binary class (positive or negative). Given the total number of samples is *m* and out of which k belongs to negative class, the method examines the expression pattern of modules across the samples and identify modules which tend to have significant association with the clinical trait of interest. The method is based on the observation that the samples of the two class will show clear distinction in distribution of ranks if they are arranged according to expression values of a gene having true differential expression. However, in module level analysis instead of a single gene we deal with set of genes assigned to the module. Therefore, in order to find sample ranking based on expression of modules as whole, a matrix with *n* rows, equal to the number of genes in the module and *m* columns, equal to the number of total samples was created. Each row of the matrix contains samples arranged in ascending order as per the expression values of genes. A position vector was created by calculating the negative class sample frequency for each column of the matrix. For a module which is not related to clinical trait, the first k largest frequencies will be uniformly distributed across the position vector. However, a module having significant relation with clinical trait will cause larger frequencies to concentrate towards one end of the position vector. The index of first k largest frequencies were summed to get a score G_s_. A significantly lower G_s_ score represents lower expression of module in negative class sample and a significantly higher score represents higher expression of module in negative class sample.

### Calculation of score G_s_

A module Μ; was defined as collection of genes g_i_(i=1...n) having similar expression pattern across the samples s_j_(j=1...m). The expression information of g_i_ε Μ across samples can be stored in a nxm matrix E where value E_ij_ represents the expression information of i^th^ gene in j^th^ sample. A sample s_j_ was assigned to set L^0^ if it belongs to negative class and to set L^1^ if belongs to positive class. The expression matrix E was converted to *nxm* index matrix where value I_ij_ was set as per Eq. (1). (see PDF)

### Assessing the significance of score G_s_

A null distribution of score G_s_ was calculated by assuming that the module is not associated with the clinical trait. In case of no association, the position vector ν will have uniform distribution of first k largest values. Therefore, in order to assess the significance of G_s_ score of a module having n number of genes following steps were performed:

[1] False modules were created by randomly selecting n genes from the total number of genes for which expression data is available.

[2] The score G_s_ for False module was calculated.

[3] Step 1 and 2 were repeated 1000 time to generate a null distribution of score G_s_.

A module with G_s_ score greater than |Μ;±(1.96xS)| was considered significantly associated with negative class samples. Where Μ; and S are mean and standard deviation of the null distribution respectively. The score G_s_ was further converted to scaled G_s_score (G_s_^score^) and he standardised G_s_score (G_s_^standardised^ ). The score G_s_was scaled to have value between -1 and +1 using Eq. (2). (see PDF)

## Results:

The R-package WGCNA [11] was used to identify set of co-expressed genes from muscle transcriptome of healthy (NGT) and diabetes (T2D) subjects [12]. In total WGCNA has identified 30 modules having tightly co-expressed genes grouped together. In order to assess the association between modules and subject diabetes status we applied the AOR and module Eigen gene (as implemented in WGCNA package) method to expression data of each of the module. Using the cutoff of p value < 0.001 thirteen modules were found significantly associated with subject diabetes status using AOR method whereas module Eigen gene method was not able to produce statistical significant result for any of the module (Figure 1 - see PDF). This showed that the subject diabetes status have significant affect on muscle gene expression.

## Discussion:

In this study, analysis of rank (AOR) method has been used to assess the association between clinical traits and co-expression modules. Application of the method on muscle gene expression profile identified significant association between module and subject diabetes status highlighting importance of AOR method in identifying hidden patterns across gene expression profiles. While interpreting the results of the AOR method both type of score G_s_^scaled^and G_s_^standardised^ should be taken into consideration along with the obtained p-value. The significant p-value obtained using AOR method for module-trait association just indicates that, more number of negative class samples, than expected by chance, are concentrated at the beginning/end of the position vector. The similar sign for G_s_^standardised^ and G_s_^scaled^score for a module supports deregulation of genes belonging to concerned module between negative and positive class samples. However, opposite signs for G_s_^standardised^and G_s_^scaled^suggest the existence of expression heterogeneity within negative class samples with respect to expression of genes belonging to concern module.

## Conclusion:

The AOR method helps in identifying hidden patterns from gene expression data and can provide deeper insights into disease biology by discovering co-expression modules linked to clinical-traits.
